# Malarial pigment haemozoin, IFN-gamma, TNF-alpha, IL-1beta and LPS do not stimulate expression of inducible nitric oxide synthase and production of nitric oxide in immuno-purified human monocytes

**DOI:** 10.1186/1475-2875-6-73

**Published:** 2007-06-02

**Authors:** Oleksii A Skorokhod, Evelin Schwarzer, Monica Ceretto, Paolo Arese

**Affiliations:** 1 Department of Genetics, Biology and Biochemistry, University of Torino Medical School, Torino, Italy

## Abstract

**Background:**

Enhanced production of nitric oxide (NO) following upmodulation of the inducible isoform of NO synthase (iNOS) by haemozoin (HZ), inflammatory cytokines and LPS may provide protection against *Plasmodium falciparum *malaria by killing hepatic and blood forms of parasites and inhibiting the cytoadherence of parasitized erythrocytes (RBC) to endothelial cells. Monocytes and macrophages are considered to contribute importantly to protective upregulation of iNOS and production of NO. Data obtained with murine phagocytes fed with human HZ and synthetic HZ (sHZ) indicate that supplemental treatment of those cells with IFN-gamma elicited significant increases in protein and mRNA expression of iNOS and NO production, providing a potential mechanism linking HZ phagocytosis and increased production of NO. Purpose of this study was to analyse the effect of *P. falciparum *HZ and sHZ supplemental to treatment with IFN-gamma and/or a stimulatory cytokine-LPS mix on iNOS protein and mRNA expression in immuno-purified human monocytes.

**Methods:**

Adherent immunopurified human monocytes (purity >85%), and murine phagocytic cell lines RAW 264.7, N11 and ANA1 were fed or not with *P. falciparum *HZ or sHZ and treated or not with IFN-gamma or a stimulatory cytokine-LPS mix. Production of NO was quantified in supernatants, iNOS protein and mRNA expression were measured after immunoprecipitation and Western blotting and quantitative RT-PCT, respectively.

**Results:**

Phagocytosis of HZ/sHZ by human monocytes did not increase iNOS protein and mRNA expression and NO production either after stimulation by IFN-gamma or the cytokine-LPS mix. By contrast, in HZ/sHZ-laden murine macrophages, identical treatment with IFN-gamma and the cytokine-LPS mix elicited significant increases in protein and mRNA expression of iNOS and NOS metabolites production, in agreement with literature data.

**Conclusion:**

Results indicate that human monocytes fed or not with HZ/sHZ were constantly unable to express iNOS and generate NOS metabolites even after stimulation with IFN-gamma or a cytokine-LSP mix that were very active on HZ-fed murine phagocytic lines. Present data do not support the hypothesis that monocytes are mediators of anti-parasitic defence in clinical malaria via activation of iNOS and production of NO, and suggest caution in extrapolating data obtained with murine or hybrid systems to human malaria.

## Background

Nitric oxide (NO) is a potent free radical produced by nitric oxide synthase (NOS) [1,2]. Activity of inducible NOS (iNOS), the high-output, cytokine-modulated isoform of NOS varies very widely in different species and cell types [3]. In humans, endothelia, hepatocytes and smooth muscle cells are the major contributing cells, while monocytes/macrophages appear to produce no or little NO in response to IFN-gamma, TNF-alpha, or IL-1beta [3-6], highly effective stimuli in murine macrophages [7]. Data indicate that severe murine and human malaria is accompanied by increased iNOS activity and production of NO [8,9], considered to be beneficial because NO was shown to kill parasites [10,11]. Secondly, elevated NO levels in the blood of malaria patients were associated with inhibition of adhesion of parasites to endothelia [12]. Increased NO levels and protection against malaria have been linked to a single nucleotide polymorphism in the promoter of iNOS gene termed NOS2^Lambaréné ^(G-954C) mutation [13]. Blood monocytes of those protected heterozygotes had a 7-fold higher baseline iNOS activity [13]. Recently, it has been shown that phagocytosis of *P. falciparum *haemozoin (HZ) and synthetic HZ (sHZ, beta-haematin) by murine phagocytes significantly increased IFN-gamma-mediated iNOS expression and NO production [14], offering a mechanistic link to the increased, possibly protective NO levels observed in clinical malaria. Here the authors analysed iNOS mRNA, protein expression and NO production in HZ/sHZ-fed immuno-purified human monocytes and were unable to find any iNOS expression and NO production even after maximal stimulation by IFN-gamma, TNF-alpha, IL-1beta and LPS, whereas these stimuli were highly effective in HZ-laden murine phagocytes. Lack of iNOS response by human monocytes shown here casts doubt on the capacity of those cells to produce NO in vivo and mediate protection in clinical malaria, and suggests caution in transferring data obtained with non-human cells to human malaria.

## Methods

### Materials

Unless otherwise stated, reagents were from Sigma Chem Co, St Louis, MO; cell culture supplements were from Invitrogen, Carlsbad, CA.

### Culture of *P. falciparum *and isolation of native HZ

*P. falciparum *parasites (Palo Alto strain, mycoplasma-free) were kept in culture as described [15]. HZ was harvested from synchronous cultures at ring stage, 12 h after infection of RBCs added to separated schizonts. To avoid contamination with schizont debris, HZ was collected from the 10–40% interphase after centrifugation at 5,000 *g *on a discontinuous Percoll-mannitol density gradient. HZ was washed at 4°C 5 times with 10 mM phosphate buffer, pH 8.0, containing 10 mM mannitol and once with phosphate buffered saline (PBS), pH 7.4, and stored at -20°C at 20% (vol/vol) in PBS. Native HZ used in this study corresponds closely to the material phagocytosed in vivo by human phagocytes.

### Preparation of beta-haematin (synthetic HZ, sHZ) and haemin

sHZ was synthesized according to the Slater *et al. *procedure [16] modified as follows. Haemin chloride (380 mg) was dissolved in 80 mL of 0.1 M NaOH and haem precipitated by addition of 28 mL glacial acetic acid for 12 h at 80°C. Crystalline sHZ was washed four times with milliQ water, four times for 3 h and two times for 1 h with 100 mM sodium bicarbonate at pH 9.1, in order to fully remove free haem, and finally four times with milliQ water. Remaining insoluble sHZ was washed once with PBS 1:5 (vol/vol), resuspended in PBS 1:5 (vol/vol) and stored at 4°C. Haemin (monomeric haem) was added to the adherent cells as met-haem-albumin, containing 2.5 mM haemin in 10 mM Tris, pH 7.5, 1.25% albumin and 100 mM NaCl. The solution was filtered through a 0.2-μm-pore filter before adding to the cells.

### Opsonization of HZ and sHZ

Immediately before phagocytosis HZ and sHZ were washed in PBS, finely dispersed in PBS at 30% (vol/vol) and added to the same volume of freshly prepared human A-serum. After 30-min incubation at 37°C, opsonization was terminated by one washing in PBS immediately before addition to adherent cells.

### Immuno-purified human monocytes

Monocytes were isolated from freshly discarded buffy coats from peripheral blood of healthy Italian donors by Ficoll centrifugation and purified by immuno-depletion of lymphocytes with Dynabeads M-450 CD2 Pan T and M-450 CD19 Pan B (Dynal, Oslo, Norway) as described [15]. By this method monocytes were 86.7 ± 3.7% pure (mean ± SE, n = 5), as judged from physical parameters and the expression of monocytic surface antigens (CD14^+^, MHC class I and II) evaluated by cytofluorimetry. The contaminating cells (approx. 14%) were CD2-negative/CD3-positive cells unable to bind to the beads to allow immunomagnetic separation. The number of iNOS-expressing NK-cells was negligible. To the best of our knowledge, there is no evidence that "contaminating" lymphocytes express iNOS or interfere with iNOS-expression in human monocytes. Abs used were: anti-CD14 (3C10; American Type Culture Collection (ATCC), Manassas, VA), anti MHC class I (W6.32, ATCC) and anti-MHC class II (BT-2.9, [17]). The immuno-purification provided higher yields of pure monocytes compared to purification by cytoadherence to plastics. Based on the same quality criteria as above, adherence-purified monocytes, analysed within 12 h from plating, were 34.2 ± 3.0% pure (mean ± SE, n = 19). Monocytes, resuspended at 1 × 10^6 ^and 5.0 × 10^6 ^cells per mL to obtain non-confluent and confluent cell condition, respectively, were plated in 35 mm-diameter culture dishes. Cells were kept either in RPMI 1640 cell culture medium, supplemented with L-glutamine, sodium pyruvate, non-essential amino acids and 10% (vol/vol) foetal calf serum (RS-FCS) or in macrophage serum-free medium (M-SFM). After 30 min incubation at 37°C dishes were washed three times with RPMI 1640 to remove non-adherent cells. Two mL of RS-FCS or M-SFM were added to each dish and phagocytosis and cytokine treatment were immediately started, as described below. Cells seeded at different densities in different media gave similar results. Adherent monocytes kept in M-SFM for two days before starting experiments showed same results as freshly plated cells. Results described here were obtained with 1 × 10^7 ^cells/dish in M-SFM plated on the day of experiment.

### Murine phagocytic cell lines

Cells of the murine phagocytic cell lines ANA1 and RAW 264.7 [18,19] and the murine microglial phagocytic monocytic cell line N11 [20] were plated at 5.0 × 10^6 ^cells (confluent cells condition) per mL RPMI 1640, containing 10% FCS, in 35 mm-diameter culture dishes and incubated for 24 h before starting phagocytosis and cytokine treatment as described before. The 24 h period after plating was shown to be adequate for efficient induction of iNOS.

### Phagocytosis and cytokine treatment of cells

Phagocytosis was started by adding opsonized HZ or opsonized sHZ at 50 RBC equivalents in terms of haem content per cell to adherent human monocytes or RAW 264.7, ANA1, and N11 murine cells. Alternatively, cells were treated with 100 μM haemin or remained untreated as controls. Portions of the phagocytosing and control cells were stimulated with 100 U/mL IFN-gamma (human recombinant, R&D, Minneapolis, MN, USA) at the beginning of phagocytosis. Maximal iNOS stimulation was elicited by treating cells with the stimulatory cytokine-LPS mix containing 400 U/mL IFN-gamma (R&D), 500 U/mL TNF-alpha (human recombinant, PreproTech, Rocky Hill, NJ), 100 U/mL IL-1beta (human recombinant, PreproTech) and 20 μg/mL LPS (from *Escherichia coli *055:B5, Sigma) (final concentrations). After 24 h incubation, supernatants were collected for assessment of NO production. The cells were washed three times with PBS and used in immunoprecipitation, Western blotting and mRNA studies.

### Viability and cytokine response of isolated monocytes

Cell apoptosis was assayed by Annexin-V binding evaluated by cytofluorimetry according to manufacturer's specifications (Alexis Biochemicals, Lausen, Switzerland). Additionally, cell death was excluded by propidium iodide staining performed according to the manufacturer's instructions. Expression of the cytokine-responsive membrane antigens MHC class II and CD54 was tested after exposure of cells to IFN-gamma and the cytokine-LPS mix [15]. Labeling and subsequent phenotypic analysis by cytofluorimetry of monocytes was performed on day 1 and 2 after cell plating. Monoclonal Abs were: anti-CD54 (Serotec, Oxford, U.K.) and anti-MHC class II (BT-2.9 hybridome [17]. Bound antibodies were revealed by FITC-conjugated F(ab') goat anti-mouse IgG. Mean fluorescence intensity (MFI) was measured and data analysed using a FACScan cytofluorograph (Becton Dickinson, Sunnyvale, CA) and CellQuest software.

### Analysis of signal transduction by the IFN-gamma receptor. Phosphorylation of JAK-2 after IFN-gamma stimulation

Adherent control and HZ-fed immuno-purified human monocytes were washed and harvested in ice-cold PBS, containing 20 mM mannitol 24 h after phagocytosis. After 10-min preincubation in PBS containing 1 mM ortho-vanadate, cells were treated with 500 U/mL IFN-gamma for 5 min on ice. Cells were lysed in 50 mM Tris-buffer, pH 7.4, containing 150 mM NaCl, 1% Triton X-100, 1 mM ortho-vanadate and complete protease inhibitor cocktail (Roche, Mannheim, Germany). Phosphorylated lysate proteins from 20 × 10^6 ^monocytes were immunoprecipitated by sequential treatment with a monoclonal anti-phosphotyrosine antibody (clone G410, Upstate Biotechnology, Lake Placid, NY) and anti-mouse IgG-agarose. Immunoprecipitates were separated in a 7% acrylamide SDS-PAGE, transferred to nitrocellulose membranes and probed with a rabbit polyclonal anti-JAK-2 antibody (Upstate Biotechnology). Enhanced chemiluminescence was used for detection of phosphorylated JAK-2.

### NO quantification in cell culture supernatants

Production of NO was assessed by measuring the accumulation of nitrite in the cell culture supernatants by the Griess reaction as described [21]. Briefly, 150 μL of centrifuged cell supernatants were mixed with the same volume of Griess reagent and the absorbance quantified at 550 nm with a Benchmark Plus plate reader (Bio-Rad Laboratories, Hercules, CA), using the Microplate Manager 5.2 software (Bio-Rad). Nitrite dissolved in the different media was used to generate standard curves for each plate reading. Protein concentration in cell lysates in each sample was assayed using the Bio-Rad protein assay according to the manufacturer's instructions.

### Immunoprecipitation of iNOS

Washed human monocytes and murine phagocytic cell lines were supplemented with complete protease inhibitor cocktail (Roche) and 5 mM N-ethylmaleimide, harvested and lysed for 10 min at 4°C in 20 mM Tris-buffer, pH 7.4, containing 150 mM NaCl, 1% (wt/vol) SDS, Complete protease inhibitor cocktail (Roche) and 1 mM ortho-vanadate. The lysate was centrifuged and the supernatant protein concentration adjusted to 1 mg/mL of the immune-precipitation (IP) buffer, composed of 10 mM Tris-buffer, pH 7.4, 1% (vol/vol) Triton X-100, 150 mM NaCl, 1 mM EDTA, 1 mM EGTA, 0.2 mM ortho-vanadate, 0.2 mM PMSF, 0.5% (vol/vol) NP-40 (final concentrations). Monoclonal anti-iNOS antibody (BD Transduction Laboratories, Lexington, KY, USA) was added at a final dilution of 1:500 for 12 h at 4°C. Antibody-bound iNOS was precipitated with anti-mouse IgG-agarose (20 μL/mL, final concentration) added for 2.5 h at 4°C and sedimented by centrifugation. After three washes with IP-buffer the immune-precipitated proteins were solubilized in SDS-sample buffer for 3 min at 95°C and frozen until use for SDS-PAGE and subsequent Western blotting.

### Western blotting of iNOS

Washed human monocytes and murine phagocytic cell lines were supplemented with Complete protease inhibitor cocktail (Roche) and 5 mM N-ethylmaleimide. Cells were then lysed in cold SDS-Sample Buffer and lysate proteins (20 μg) or immunoprecipitated proteins (originating from 500 μg lysate protein) were subjected to SDS-PAGE, 10% acrylamide (wt/vol), and separated proteins were transferred onto a polyvinylidene difluoride membrane (PVDF; Millipore, Bedford, MA). After the transfer the membrane was rinsed in methanol for 20 s, and than incubated with monoclonal mouse anti-iNOS antibody (BD Transduction Laboratories) in PBS with 1% (wt/vol) BSA for 1 h. Proteins were detected with an anti-mouse HRP-conjugated sheep antibody (Amersham Biosciences, Buckinghamshire, UK) and visualized subsequently by ECL. Recombinant iNOS (Sigma) was used as positive control.

### RT-PCR studies

The expression of human and murine iNOS mRNA was analysed by quantitative real-time RT-PCR and qualitative RT-PCR. Total cellular RNA was quantitatively isolated from washed human monocytes and murine phagocytic cell lines using Standard RNA Releaser, RNA specific resin and elution solution (RNA Extraction Kit, Nurex, Sassari, Italy) following the manufacturer's specifications. Subsequently, the RNA was reverse-transcribed using Moloney murine leukemia virus reverse transcriptase (7.7 U/μL final concentration; Invitrogen) and oligo-dT (11.5 μg/μL final concentration; Invitrogen). PCR was performed using cDNA from 50,000 cells with primer pairs specific for iNOS.

### Quantitative real-time RT-PCR

For human iNOS two different primer pairs were used. The first one was 5'-CAGCGGGATGACTTTCCAA-3' and 5'-AGGCAAGATTTGGACCTGCA-3' [22,23]. The second one was 5'-AGCGGGATGACTTTCCAAGA-3' and 5'-TAATGGACCCCAGGCAAGATT-3' [24]. GAPDH intron overlappingprimers were from the Bio-Rad library (Bio-Rad). Murine iNOS primer pair was 5'-TGCCCCTTCAATGGTTGGTA and 5'-ACTGGAGGGACCAGCCAAAT-3'. Murine beta-actin primers were 5'-TCACCCACACTGTGCCCATCTACGA-3' and 5'-GGATGCCACAGGATTCCATACCCA-3' [18]. For each 50 μL real-time RT-PCR reaction mix, 2.0 μL cDNA (corresponding to 50,000 cells), 1.0 μL sense and anti-sense primer (both 10 μM; Invitrogen), 1.0 μL dNTP (10 mM; Applied Biosystem, Foster City, CA), 3.0 μL MgCl2 (50 mM), 2.5 U Platinum Taq DNA Polymerase (Invitrogen), 5.0 μL Buffer (10×), 3.25 μL SYBR Green (final dilution 1:10,000), and 33.25 μL PCR-grade water were mixed. After denaturation for 2 min at 94°C, RT-PCR assays were carried out for 50 cycles with denaturation for 30 s at 94°C, annealing for 30 s at 60°C, and extension for 30 s at 72°C. The specified mode of reaction was controlled with the melting curve. Real-time RT-PCR analyses were performed with the iCycler iQ real-time RT-PCR detection system (Bio-Rad).

### Qualitative RT-PCR

Qualitative RT-PCR was performed with MyCycler detection system (Bio-Rad) with the same reaction mix and temperature conditions used for real-time RT-PCR. For murine iNOS primers were 5'-GGAGATCAATGTGGCTGTGC-3' and 5'-AAGGCCAAACACAGCATACC-3' (631 bp); primers for β-actin were 5'-GGTCATCTTCTCGCGGTTGGCCTTGGGGT-3' and 5'-CCCCAGGCACCAGGGCGTGAT-3' (230 bp) [20].

### Statistical analysis

Data are presented as mean ± standard error of the mean (SE). Statistical significance was analysed using two-sample *t*-test.

## Results

### Viability and cytokine response of immuno-purified human monocytes

**Figure 1:**
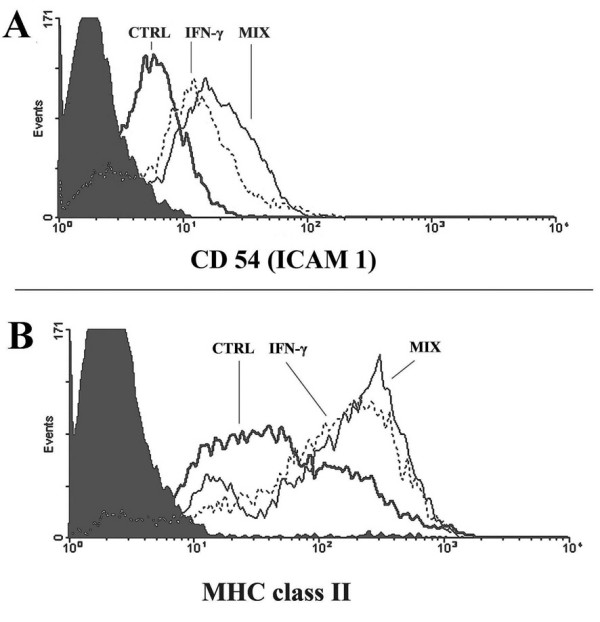
**Expression profiles of CD54 (A) and MHC class II (B) on adherent immuno-purified human monocytes**. One representative experiment shows the upregulation of cell surface antigenes by IFN-gamma (dotted line) or cytokine-LPS mix (solid thin line) 48 h after stimulation compared to untreated controls (solid thick line) measured by flow cytometry. Background is plotted as solid area.

**Figure 2:**
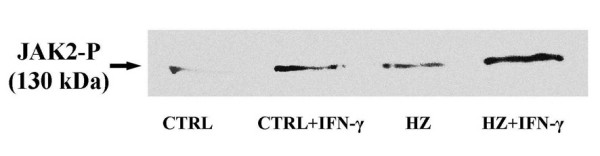
**HZ-fed immuno-purified human monocytes transmit the IFN-gamma signal into the cell with subsequent JAK2 phosphorylation**. Adherent monocytes were fed or not with native *P. falciparum *HZ for 24 h. Phosphorylated JAK-2 from control and HZ-fed monocytes treated or not with IFN-gamma was immunoprecipitated with anti-phosphotyrosine and probed with anti-JAK-2 in Western blot. One representative experiments out of 3 with similar results.

Monocytes viability was tested by Annexin-V binding. The percentage of AnnexinV-binding-cells (considered to be apoptotic) was negligible during the experimental period and never higher than 4% at 48 h after plating in control, HZ or sHZ-fed and IFN-gamma and cytokine-LPS mix-treated monocytes. The percentage of dead cells positive to propidium iodide staining was lower than 2%. Human monocytes respond to stimulation by LPS, IFN-gamma and inflammatory cytokines with upregulation of membrane expression of CD54 (ICAM 1) and MHC class II [15,25]. Ability of immuno-purified human monocytes (purity >85%) to respond to LPS and inflammatory cytokines was tested after treatment with IFN-gamma (100 U/mL) or a cytokine-LPS mix containing IFN-gamma (400 U/mL), TNF-alpha (500 U/mL), IL-1beta (100 U/mL) and LPS (20 μg/mL) (final concentrations). Monocytes were responsive to cytokines, as membrane expression of CD54 (ICAM 1) and MHC class II was progressively increased at 24 and 48 h after start of treatment. At 48 h, stimulation by IFN-gamma or cytokine-LPS mix increased surface expression of CD54 1.4 ± 0.1-fold and 1.8 ± 0.5-fold (n = 5), respectively, and surface expression of MHC class II 1.9 ± 0.4-fold and 2.2 ± 0.4-fold (n = 5), respectively (Figure 1). HZ-fed monocytes were shown to be less responsive to IFN-gamma in terms of increased expression of surface antigen [15]. However, IFN-gamma receptor and trans-membrane signal transduction path were apparently intact in HZ-fed monocytes, as indicated by the normal phosphorylation of JAK-2 (Figure 2).

### Effect of IFN-gamma and cytokine-LPS mix on NO production by immuno-purified human monocytes and murine phagocytic cell lines fed or not with HZ and sHZ

**Figure 3:**
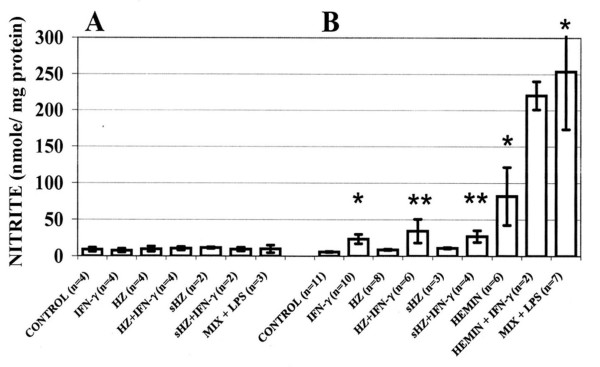
**NO production by (A) immuno-purified human monocytes and (B) RAW264.7 murine macrophages**. Cells were fed or not with HZ or sHZ, or treated with haemin added as met-haem-albumin. A portion of the cells was additionally stimulated with IFN-gamma (100 U/mL) or a stimulatory cytokine-LPS mix (MIX+LPS) containing IFN-gamma (400 U/mL), TNF-alpha (500 U/mL), IL-1beta (100 U/mL) and LPS (20 μg/mL) (final concentrations). After 24 h incubation, NO production was measured as nitrite in the cell culture supernatants and expressed as nmole/mg cell protein. Mean values ± SE (n = 2–11). The difference between untreated/unfed controls and treated/fed cells was tested for significance:*p < 0.05; **p < 0.1.

Stimulation by IFN-gamma (100 U/mL) or by the cytokine-LPS mix did not increase NO production (measured as nitrite) in HZ- or sHZ-fed, haemin treated or unfed control human monocytes (Figure 3A). By contrast, murine RAW 264.7 macrophages treated with IFN-gamma (100 U/mL) for 24 h showed a significant 4.1-fold increase in the production of NO compared to untreated controls. NO in the supernatant of IFN-gamma-treated murine macrophages attained 23.4 nmoles/mg protein (Figure 3B). Stimulation of HZ-fed or haemin-treated murine macrophages with IFN-gamma (100 U/mL) further enhanced NO production 6-fold and 2.6-fold, respectively, compared to HZ-fed or haemin-treated but IFN-gamma untreated cells (Figure 3B). Feeding with sHZ led to lower IFN-gamma-mediated stimulation of NO production compared to HZ. Remarkably, treatment of unfed murine RAW 264.7 macrophages with the stimulatory cytokine-LPS mix increased NO output up to 44.3-fold. (Figure 3B). Similar results were obtained with murine phagocytic lines ANA1 and N11 (results not shown).

### Effect of IFN-gamma and cytokine-LPS mix on iNOS protein expression in immuno-purified human monocytes and murine phagocytic cell lines fed or not with HZ and sHZ

**Figure 4:**
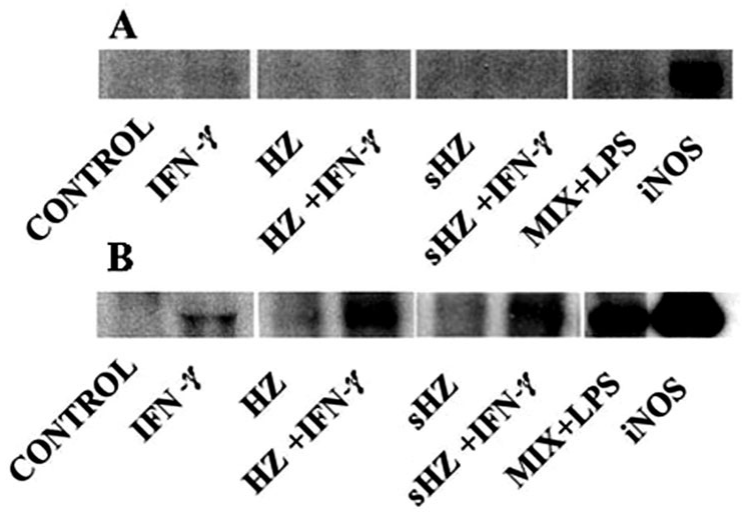
**iNOS protein expression in (A) immuno-purified human monocytes and (B) RAW264.7 murine macrophages**. Cells were fed or not with HZ or sHZ. A portion of the cells was additionally stimulated with IFN-gamma (100 U/ml) or a stimulatory cytokine-LPS mix (MIX+LPS) containing IFN-gamma (400 U/mL), TNF-alpha (500 U/mL), IL-1beta (100 U/mL) and LPS (20 μg/mL) (final concentrations). After 24 h incubation cells were analysed for iNOS expression. Immune-precipitated iNOS from lysate proteins of human monocytes (A) or lysate proteins of RAW264.7 murine macrophages (B) were separated by 10% SDS-PAGE and blotted to PVDF. iNOS was visualized via ECL by binding of a monoclonal anti-iNOS antibody. Recombinant iNOS (Sigma) was used as positive control. Blots shown are representative for five separate experiments.

To detect even very low copy numbers of iNOS in human monocyte lysates, iNOS was concentrated by immunoprecipitation before Western blot analysis. No iNOS was detectable in HZ or sHZ fed or unfed human monocytes even after enrichment or addition of the stimulatory cytokine-LPS mix that strongly enhanced expression of iNOS in murine RAW 264.7 macrophages, detectable already without enrichment by immunoprecipitation (Figure 4A and Figure 4B). Murine cells fed with sHZ were less responsive compared to cells fed with native HZ. Overall, there was a parallelism between protein expression and NO production (Figure 3B). Similar results were obtained with murine phagocytic cell lines ANA1 and N11 (results not shown).

### Effect of IFN-gamma and cytokine-LPS mix on iNOS mRNA expression in immuno-purified human monocytes and murine phagocytic cell lines fed or not with HZ and sHZ

**Figure 5:**
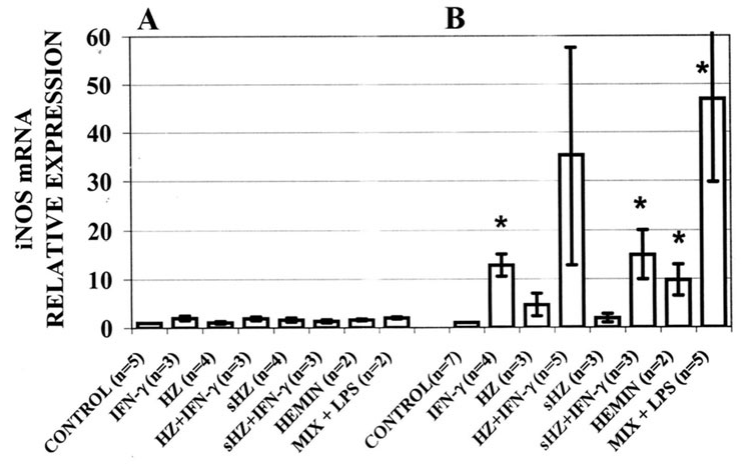
**iNOS mRNA expression in (A) immuno-purified human monocytes and (B) RAW264.7 murine macrophages**. Cells were fed or not with HZ or sHZ, or treated with haemin added as met-haem-albumin. A portion of the cells was additionally stimulated with IFN-gamma (100 U/mL) or a stimulatory cytokine-LPS mix (MIX+LPS) containing IFN-gamma (400 U/mL), TNF-alpha (500 U/mL), IL-1beta (100 U/mL) and LPS (20 μg/mL) (final concentrations). After 24 h incubation, mRNA was extracted from cells and iNOS-specific mRNA was quantified by real-time RT-PCR. Data are presented as fold increase in iNOS-mRNA level vs control. Mean values ± SE (n = 2–7). The difference between untreated/unfed controls and treated/fed cells was tested for significance: *p < 0.05.

Again, iNOS mRNA expression in human monocytes was totally unresponsive to any stimulus applied (Figure 5A) while in murine phagocytic cell lines IFN-gamma and the stimulatory cytokine-LPS mix induced remarkable increases in iNOS-specific mRNA levels detected by qualitative RT-PCR (data not shown) and confirmed by real-time RT-PCR (Figure 5B). In murine RAW 264.7 macrophages, association of IFN-gamma treatment with phagocytosis of HZ or sHZ enhanced iNOS mRNA expression 35.2- and 15-fold, respectively. To exclude artefacts due to unspecific effects of haemin possibly liberated in the phagocytes from ingested sHZ, the effect of haemin on iNOS mRNA expression was also checked, providing negative results in human monocytes and positive results in murine cells (Figure 5A and Figure 5B). The quantitative discrepancy between the modest iNOS mRNA induction and the very high NO production elicited by haemin in murine cells (Figure 3B), could be explained by the unspecific reactivity of the Griess reagent with bilirubin produced by macrophages (E. Schwarzer, unpublished observation). Very similar results were obtained with murine phagocytic cell lines ANA1 and N11 (results not shown).

## Discussion

Human and murine phagocytic cell lines ingest large amounts of HZ that upsets several functions connected with modulation of immunity. HZ-laden human monocytes were unable to kill ingested pathogens and produce oxidative burst [26,27], did not upregulate MHC class II and CD54 (ICAM-1) after IFN-gamma stimulation [15], produced increased amounts of TNF-alpha and IL-1beta [28] while HZ-laden human and murine monocytes were severely impaired in their ability to differentiate and mature to functional dendritic cells [29,30]. Recently, phagocytosis of *falciparum *HZ and sHZ by murine phagocytes was shown to significantly increase IFN-gamma-mediated iNOS expression and NO production [14]. Those data have prompted present work aimed to check transferability of observations obtained with murine phagocytes fed with human HZ to a fully human system formed by immuno-purified human monocytes fed with native *falciparum *HZ. A second aim was to validate the role of HZ phagocytosis by monocytes as inducer of increased iNOS expression and NO levels observed in clinical malaria [31]. Present data obtained with HZ-fed murine phagocytes as the target cells confirm results obtained by Jaramillo *et al. *[14] with a similar experimental system. By contrast, no induction of iNOS protein or mRNA expression, and no production of NO were observed in HZ-fed immuno-purified human monocytes even after maximal stimulation by an appropriate stimulatory cytokine-LPS mix. These negative results are in line with studies indicating that human monocytes are unable to express iNOS even after maximal cytokine/LSP stimulation [3-5], but disagree with a number of other studies [6,9,13,31]. Of note, present results were obtained with immuno-purified monocytes while the vast majority of data showing increased iNOS espression in malaria were obtained with monocytes isolated by gradient centrifugation and adhesion to plastics. Present group's experience indicates that the latter method produces monocytes heavily contaminated with iNOS-expressing lymphocytes.

Apart from these methodological problems, there is no general acceptance as to the importance of iNOS and NO in malaria. For example, in a recent review [32], evidence was summarized indicating that killing of *Plasmodium *by NO in vivo is unlikely, protective role of NO controversial and overproduction of NO not sufficiently supported. In fact, at variance with studies in Africa [8,9] studies performed in Papua New Guinea [33] found no relationship between NO levels and parasitaemia either cross-sectionally at baseline or comparing the same subjects during parasitaemic and non-parasitaemic episodes. In those studies, iNOS expression and activity in monocytes were not related either to parasitaemia or to iNOS metabolite levels [33]. Present data showing lack of response of iNOS expression and NO production by stimulated HZ-fed human monocytes, agree with this critical attitude, cast serious doubt on the capacity of human monocytes to generate NO, and suggest caution in the frequent extrapolation to human malaria of data obtained with murine or hybrid systems. Future studies performed with highly purified blood monocytes will confirm or disprove available data on NO protection in malaria, and will clarify whether different ethnic backgrounds (Africa vs Papua New Guinea) may explain divergent iNOS expression and modulation.

## Authors' contributions

OAS and ES designed the research, performed the experiments and wrote the paper. MC helped with the experiments. PA helped design the research, examined and interpreted the data and helped write the paper.
